# Improved Adaptive Augmentation Control for a Flexible Launch Vehicle with Elastic Vibration

**DOI:** 10.3390/e23081058

**Published:** 2021-08-16

**Authors:** Aiping Pang, Hongbo Zhou, Wenjie Cai, Jing Zhang

**Affiliations:** 1 College of Electrical Engineering, Guizhou University, Guiyang 550025, China; appang@gzu.edu.cn (A.P.); hit_zhb@163.com (H.Z.); 2 Guizhou Provincial Key Laboratory of Internet + Intelligent Manufacturing, Guiyang 550025, China; 3 China Academy of Launch Vehicle Technology, Beijing 100076, China; caiwenjie217@126.com

**Keywords:** multiplicative adaptation, gain adjustment, spectral damping, robust stability

## Abstract

The continuous development of spacecraft with large flexible structures has resulted in an increase in the mass and aspect ratio of launch vehicles, while the wide application of lightweight materials in the aerospace field has increased the flexible modes of launch vehicles. In order to solve the problem of deviation from the nominal control or even destabilization of the system caused by uncertainties such as unknown or unmodelled dynamics, frequency perturbation of the flexible mode, changes in its own parameters, and external environmental disturbances during the flight of such large-scale flexible launch vehicles with simultaneous structural deformation, rigid-elastic coupling and multimodal vibrations, an improved adaptive augmentation control method based on model reference adaption, and spectral damping is proposed in this paper, including a basic PD controller, a reference model, and an adaptive gain adjustment based on spectral damping. The baseline PD controller was used for flight attitude control in the nominal state. In the non-nominal state, the spectral dampers in the adaptive gain adjustment law extracted and processed the high-frequency signal from the tracking error and control-command error between the reference model and the actual system to generate the adaptive gain. The adjustment gain was multiplied by the baseline controller gain to increase/decrease the overall gain of the system to improve the system’s performance and robust stability, so that the system had the ability to return to the nominal state when it was affected by various uncertainties and deviated from the nominal state, or even destabilized.

## 1. Introduction

As the exploration of the space environment progresses, the missions of spacecraft exploration become more and more diversified, and as the application of polymer materials in the space field progresses, the structure of spacecraft is gradually developing towards large and flexible structures. In order to carry these large and flexible-structure spacecraft, launch vehicles with a large carrying capacity have become an inevitable requirement of space-development strategies [1]. At the same time, the lightweight polymer material used in the body of launch vehicles has increased the flexible mode of the vehicles, leading to the presence of structural deformation, rigid body-elastic vibration coupling, multi-modal vibration, and other characteristics of the body at the same time. These factors make the attitude control of launch vehicles subject to oscillations and difficult to attenuate, or even lead to system instability, which poses a new challenge to the reliability and robustness of launch vehicles [2].

For high-risk aerospace applications, both government and industry rely heavily on classical control theory, and gain-scheduling PID control is still the mainstream control method for current launch vehicles, due to the advantages of its simple structure, good anti-interference, and ease of analysis in the time domain (or frequency domain). Typical applications include the Saturn V and Space Shuttle of the United States, the Ariane of Europe, and the Long March series of launch vehicles of China [1]. The application of classical control theory in launch vehicles has matured, and is well verified with few failures. Although the classical control methods can usually meet the flight requirements, the traditional gain-scheduling PID control is no longer able to meet the control requirements of launch vehicles due to their increasing mass and aspect ratio, the increase in the flexible structure of their components, and the obvious influence of the flexible mode and elastic vibration of large launch vehicles, and is unable to cope with the control instability problems caused by the excessive interference and modal uncertainty during the flight. Due to the unknown and unmodeled dynamics, external perturbations of the flight environment, and changes in its own parameters, the attitude of a large flexible launch vehicle will inevitably generate errors during flight [3]. Traditional controllers are usually designed with high gain to suppress attitude errors, but excessive gain during full flight will easily cause the control commands to vibrate, and the flexible mode will also generate a series of vibration signals, which will affect the control effect. There are two main solutions to the problem of elastic-vibration suppression in the design of launch vehicle control systems: one is to design controllers for different characteristics using robust control theory, and the second is to suppress the elastic vibration signal by designing a notch filter. Unlike the robust controller design, the notch filter does not require significant changes to the original rocket control system to deal with elastic vibration. The zero point of the notch filter is used to eliminate the high-frequency pole of the elastic rocket system and determine the frequency center according to the system requirements of the filter. The method of using a notch filter to suppress the elastic vibration of a projectile is widely used in the design of the launcher attitude-control system. The determination of the parameters of the notch filter is the key to the design, and the location of the zero point of the notch filter; i.e., the frequency center, can be determined after the transfer function of the elastic launch vehicle is determined with sufficient accuracy in the model [4,5]. In order to solve the low-frequency, dense-frequency elastic vibration modes appearing in the launch vehicle, some scholars adopted the method of attitude control of the flexible launch vehicle by adaptive control of the adaptive notch, and the adaptive controller of the adaptive notch filter successfully stabilized the uncertain and time-varying equations of the launch vehicle model dynamics through thrust vector control [6]. Another scholar designed a bending mode filter for the whole system, which had a better filtering function for low-frequency, dense-frequency modes, and achieved good control results [7].

In response to the limitations of the classical approach, in order to increase the robust stability of the launch vehicle attitude-control system, many scholars began to work on advanced control methods, and since 1990, NASA has developed a variety of launch vehicle control techniques in the Advanced Guidance Control program, including trajectory linearization control methods, neural network adaptive control methods, and higher-order sliding-mode control methods; the development of such advanced controls has the potential to improve system performance and increase robustness [8,9,10]. Classical adaptive-control concepts were proposed for attitude-control systems applied to rockets [11,12,13]. However, many adaptive-control concepts are not feasible when applied to high-risk aerospace systems due to the stringent flight environment. In addition, many adaptive techniques appearing in the literature are not applicable to conditionally stable systems with complex flexible dynamics. Therefore, researchers have optimized model-referenced adaptive control for these situations and proposed an Adaptive Augmentation Control (AAC) [14], widely used in launch-vehicle and missile-longitudinal control in recent years. AAC a gain-adjustment method based on a model-referenced adaptive-control design that generates adaptively adjusted gain from the generalized error between the reference model and the actual system as a supplement to the nominal controller. Orr et al. introduced a scheme for adaptive control of multiplicity applicable to rockets [3], and then improved the adaptive-control scheme [15] to improve the performance of the original method with higher sensitivity to external inputs. The method was developed by the NASA Marshall Space Center (MSFC) and became a major part of the U.S. Space Launch System (SLS) to adapt to unpredictable external environmental disturbances and a variety of flight dynamics characteristics (elastic vibration of flexible modes, control structure coupling, servo delay, etc.) and to reduce the probability of flight destabilization [15,16]. NASA included the method in the development of the flight control system for the SLS program in early 2013, and tested the designed method in the F/18-A to verify the resilience of the control system in adverse flight conditions [17,18]. Brinda et al. performed an adaptive gain-adjustment controller design for the longitudinal channel of a two-stage launch vehicle using a Chebyshev high-pass filter to improve the problem of insufficient amplitude of the low-frequency part of the control signal in the original adaptive gain-adjustment structure of the low-order high-pass filter [19,20]; however, there were equal-amplitude ripples in the passband of the Chebyshev filter. Zhang applied a fault-tolerant control method and adaptive vibration frequency recognition method to AAC, and designed a corresponding correction network based on an SMM algorithm to identify each order vibration frequency to improve system control performance and stability [21]. Cui Naigang et al. applied an interference compensation control loop and active load reduction control loop based on the dilated state observer to the adaptive gain adjustment structure, and performed a simulation analysis of the pitch channel control [22]. To enhance the robustness to changes in elastic modal parameters, Domenico Trotta integrated the AAC control architecture with adaptive notch filters and proposed two novel and effective tuning methods for adaptively enhanced control systems, which were optimized by robust design and solved by genetic algorithms to achieve continuous improvement in the performance and robustness of standard launch vehicle single-axis attitude controllers in atmospheric flight [6,23]. Diego Navarro designed two adaptive augmentation control laws using a robust control design (structured H∞ control) as a baseline controller to improve the robust performance of AAC control, while analyzing the effect of the adaptive action on the classical stability margin, and validated this analysis using nonlinear time-domain stability margin evaluation techniques [24]. However, if the expansion state observer is not properly selected, the observer is easily affected by the noise signal, or even diverges when there is additional measurement noise caused by elastic vibration and other additional dynamics. However, advanced nonlinear stabilization techniques to reduce the error by increasing the control gain are not feasible for aerospace systems with high complexity; meanwhile, the above-mentioned adaptive gain-adjustment control scheme features complicated algorithms, costly computation, and challenging implementation, which introduce unknown risks to the actual system.

In this paper, we aim to establish a rigid-bullet coupling model of a large flexible spacecraft with second-order vibration signals and design an improved adaptive augmentation control method based on the reference model adaptive control method to address additional dynamics issues such as increased attitude-tracking errors and flexible-mode elastic vibrations caused by uncertainty (modeling uncertainty, frequency perturbation of the flexible mode) and external environmental interference during ascent of a large launch vehicle with a flexible mode. The scheme first determines the adjustment threshold of forward gain on the basis of the baseline PD controller, and takes the tracking error and control command error signals as the input of the two channels of the adaptive control law. The spectral dampers in the two channels (tracking error and control command elastic vibration) process the error signals (the high-pass filter extracts the high-frequency signal of a specific frequency from the error signal, and the low-frequency filter lowers the frequency to reduce the influence of the high-frequency signal on the actuator) to produce the corresponding suppression gain (error-suppression gain and elastic-suppression gain) to form the overall gain of the AAC, and increase or decrease the forward gain of the system to improve the control performance of the system. The AAC controller will not affect the PD controller when the basic PD controller is able to handle the control tasks better [3,15], whereas the AAC controller will adjust the adaptive gain to achieve the overall gain of the system when the impact of external perturbations and uncertainties is significant, so as to recover the system performance when the system deviates severely from the nominal state and meet the performance requirements of attitude control and robust stability of the large flexible launch vehicle in the flight process.

This paper is organized as follows. Section 2 introduces the rigid-bullet coupling model of a large flexible launch vehicle with a second-order elastic vibration signal. Section 3, on controller design, details the improved adaptive augmented control scheme: (1) based on the rigid-bullet coupling model of a large flexible launch vehicle, the base-line PD controller is given and the maximum critical value of the forward gain tunable is determined after analyzing the flexible mode of the system in the frequency domain; (2) Two error signals are selected between the reference model and the actual system as input signals for the two channels of the adaptive control law, thereby increasing/decreasing the overall forward gain of the system; and (3) we introduce the role of the spectrum damper and parameter selection (mainly the extraction of high-frequency signal and low-frequency output for the error input signal of two channels). Section 4 presents a simulation analysis of the improved adaptive augmentation control designed in this paper, and the simulation results of several launch vehicle runaway scenarios are presented and discussed. By comparing the traditional PD control with the adaptive augmentation controller containing the baseline PD controller, we observed that the adaptive augmentation control improved the performance and robust stability of the large flexible launch vehicle during flight.

## 2. Mathematical Model of the Launch Vehicle with Second-Order Vibration Modes

The coupling problem between the rigid body motion and elastic vibration of large launch vehicles is more prominent than that of medium-sized and small launch vehicles. The motion process is more complicated due to the large mass of large launch vehicles, the increase in the aspect ratio, and the complex interference and uncertainty during flight, so the elastic vibration mode cannot be neglected. The rigid-bullet coupling mathematical model of the launch vehicle was established based on the forces (gravity, aerodynamic, thrust, control, etc.), moments, and vibration factors during the ascent of the launch vehicle.

In the velocity coordinate system, the translational and rotational motion of the launch vehicle around the centroid (pitch channel) can be expressed as (1) and (2). Considering the plane bending vibration and torsional elastic vibration of the arrow body, the general elastic vibration equation can be obtained by using the modal superposition method and the orthogonality of the vibration pattern, as shown in (3).

(1) $$\begin{array}{ll} mV\dot{\theta }\cos\sigma = & -mg\cos\theta +qS_{m}C_{y}^{\alpha }\alpha -qS_{m}\sum_{i=1}^{n}\left(q_{iy}\int_{x_{0}}^{x_{l}}C_{y}^{\alpha }{}_{x}R_{iz}(x)dx\right)+\frac{qS_{m}}{V}C_{y}^{\alpha }(x_{d}-x_{z})\omega_{z}\\ & -\frac{qS_{m}}{V}\sum_{i=1}^{n}\left({\dot{q}}_{iy}\int_{x_{0}}^{x_{l}}C_{y}^{\alpha }{}_{x}U_{iy}(x)dx\right)+P\sin\alpha +\frac{P}{2}\delta_{\varphi }\cos\alpha -P\cos\alpha \sum_{i=1}^{n}R_{iz}(x_{R})q_{iy}+2m_{R}l_{R}{\ddot{\delta }}_{\varphi }+F_{y} \end{array}$$

(2) $$\begin{array}{ll} J_{z}{\dot{\omega }}_{z}+(J_{y}-J_{x})\omega_{x}\omega_{y}= & -2m_{R}l_{R}(x_{R}-x_{z}){\ddot{\delta }}_{\varphi }-2m_{R}l_{R}{\dot{W}}_{x}\delta_{\varphi }-\frac{P}{2}(x_{R}-x_{z})\delta_{\varphi }-P\sum_{i=1}^{n}U_{iy}(x)q_{iy}\\ & -P(x_{R}-x_{z})\sum_{i=1}^{n}R_{iz}(x)q_{iy}-qS_{m}C_{y}^{\alpha }(x_{d}-x_{z})\alpha +qS_{m}\sum_{i=1}^{n}\left(q_{iy}\int_{x_{0}}^{x_{l}}C_{y}^{\alpha }{}_{x}(x-x_{z})R_{iz}(x)dx\right)\\ & -\frac{qS_{m}}{V}m_{z}^{{\bar{\omega }}_{z}}l^{2}\omega_{z}+\frac{qS_{m}}{V}\sum_{i=1}^{n}\left({\dot{q}}_{iy}\int_{x_{0}}^{x_{l}}C_{y}^{\alpha }{}_{x}(x-x_{z})U_{iy}(x)dx\right)+M_{z} \end{array}$$

(3) $$\begin{array}{ll} {\ddot{q}}_{iy}+2\xi_{i}\omega_{i}{\dot{q}}_{iy}+\omega_{i}^{2}q_{iy}= & \left(-P\sum_{j=1}^{n}R_{jz}(x)q_{jy}+\frac{P}{2}\delta_{\varphi }\right)U_{iy}(x_{R})+2m_{R}l_{R}{\ddot{\delta }}_{\varphi }U_{iy}(x_{R})\\ & +\left(2m_{R}l_{R}{\ddot{\delta }}_{\varphi }+2m_{R}l_{R}{\dot{W}}_{x}\delta_{\varphi }\right)R_{iz}(x_{R})+qS_{m}\left(\int_{x_{0}}^{x_{l}}C_{y}^{\alpha }{}_{x}U_{iy}(x)dx\right)\alpha \\ & -qS_{m}\sum_{j=1}^{n}\left(q_{jy}\int_{x_{0}}^{x_{l}}C_{y}^{\alpha }{}_{x}U_{iy}(x)R_{jz}(x)dx\right)+\frac{qS_{m}}{V}\left(\int_{x_{0}}^{x_{l}}C_{y}^{\alpha }{}_{x}(x-x_{z})U_{iy}(x)dx\right)\omega_{z}\\ & -\frac{qS_{m}}{V}\sum_{j=1}^{n}\left({\dot{q}}_{jy}\int_{x_{0}}^{x_{l}}C_{y}^{\alpha }{}_{x}U_{iy}(x)U_{jy}(x)dx\right) \end{array}$$

The identification and meaning of the parameters in the above formula are shown in Table 1.

The nominal controller of the control system of the large flexible launch vehicle designed in this paper was based on a small perturbation linearization model, so the mathematical model of the rigid-bullet coupling of the large flexible launch vehicle was simplified to a small perturbation linearization model, as in (4) [25,26]:

(4) $$\begin{array}{l} \Delta \dot{\theta }=c_{1}\Delta \alpha +c_{2}\Delta \theta +c_{3}\delta_{\varphi }+c_{3}{}^{\prime \prime }{\ddot{\delta }}_{\varphi }+\sum_{i=1}^{n}c_{1i}{\dot{q}}_{i}+\sum_{i=1}^{n}c_{2i}q_{i}+{\bar{F}}_{y}\\ \Delta \ddot{\varphi }+b_{1}\Delta \dot{\varphi }+b_{2}\Delta \alpha +b_{3}\delta_{\varphi }+b_{3}{}^{\prime \prime }{\ddot{\delta }}_{\varphi }+\sum_{i=1}^{n}b_{1i}{\dot{q}}_{i}+\sum_{i=1}^{n}b_{2i}q_{i}={\bar{M}}_{z}\\ \Delta \varphi =\Delta \theta +\Delta \alpha \\ {\ddot{q}}_{iy}+2\xi_{i}\omega_{i}{\dot{q}}_{iy}+\omega_{i}^{2}q_{iy}=D_{1i}\Delta \dot{\varphi }+D_{2i}\Delta \alpha +D_{3i}\delta_{\varphi }+D_{3i}{}^{\prime \prime }{\ddot{\delta }}_{\varphi }+\sum_{j=1}^{n}D_{ij}{\dot{q}}_{jy}+\sum_{j=1}^{n}D_{ij}q_{jy} \end{array}$$

In addition, the period of the arrow body centroid motion is much longer than the period of the pitch attitude angle motion, so the impact of the arrow body centroid motion can be ignored in the study of the arrow body attitude angle motion, and when also ignoring the influence of each oscillation pattern in the elastic vibration equation, then (4) can be further simplified as follows:

(5) $$\begin{array}{l} \Delta \ddot{\varphi }+b_{1}\Delta \dot{\varphi }+b_{2}\Delta \varphi +b_{3}\delta_{\varphi }+\sum_{i=1}^{n}b_{1i}{\dot{q}}_{i}+\sum_{i=1}^{n}b_{2i}q_{i}={\bar{M}}_{z}\\ \Delta \varphi =\Delta \alpha \\ {\ddot{q}}_{iy}+2\xi_{i}\omega_{i}{\dot{q}}_{iy}+\omega_{i}^{2}q_{iy}=D_{1i}\Delta \dot{\varphi }+D_{2i}\Delta \varphi +D_{3i}\delta_{\varphi }+{\bar{Q}}_{iy} \end{array}$$

where ${\bar{Q}}_{iy}$ is the generalized disturbance force on the elastic vibration of the ith order.

When the attitude angle and angular rate signals of the arrow body are obtained through the attitude measuring element, there is an additional elastic vibration signal in the obtained measurement signal influenced by the elastic vibration of the arrow body, and the actual measurement signal is as follows (6):

(6) $$\begin{array}{l} \Delta \varphi_{T}=\Delta \varphi -\sum_{i}{W^{\prime }}_{i}(x_{T})q_{i}\\ \Delta {\dot{\varphi }}_{gT}=\Delta \dot{\varphi }-\sum_{i}{W^{\prime }}_{i}(x_{gT}){\dot{q}}_{i} \end{array}$$

where ${W^{\prime }}_{i}(x_{T})$ is the slope of the ith-order oscillation pattern of the attitude angular measuring element at the mounting position $x_{s}$, and ${W^{\prime }}_{i}(x_{gT})$ is the slope of the ith order vibration pattern at the installation of the attitude angular rate measurement element.

In this paper, we considered the design of a nominal controller for a second-order elastic vibration model of a launch vehicle, with the input, state variable, and output defined as:

$$u=\delta_{\varphi },\;y={\left[\begin{array}{cc} \Delta \varphi & \Delta \dot{\varphi } \end{array}\right]}^{Τ},\;x={\left[\begin{array}{cccccc} \Delta \varphi & \Delta \dot{\varphi } & q_{1} & q_{2} & {\dot{q}}_{1} & {\dot{q}}_{2} \end{array}\right]}^{Τ}$$

Here we first ignore the effect of external perturbations and build the standard state space model. External perturbations will be added to the control input signal and explained in Section 4. The state space of the system is described by (7):

(7) $$\begin{array}{l} \dot{x}=Ax+Bu\\ y=Cx+Du \end{array}$$

where the matrix A,B,C,D is given by (1)–(5),

$$A=\left[\begin{array}{cccccc} 0 & 1 & 0 & 0 & 0 & 0\\ -b_{2} & -b_{1} & -b_{21} & -b_{22} & -b_{11} & -b_{12}\\ 0 & 0 & 0 & 0 & 1 & 0\\ 0 & 0 & 0 & 0 & 0 & 1\\ D_{21} & D_{11} & -\omega_{1}^{2} & 0 & -2\xi_{1}\omega_{1} & 0\\ D_{22} & D_{12} & 0 & -\omega_{2}^{2} & 0 & -2\xi_{2}\omega_{2} \end{array}\right]B=\left[\begin{array}{c} 0\\ -b_{3}\\ 0\\ 0\\ D_{31}\\ D_{32} \end{array}\right]\;C=\left[\begin{array}{cccccc} 1 & 0 & -{W^{\prime }}_{1}\left(X_{T}\right) & -{W^{\prime }}_{2}\left(X_{T}\right) & 0 & 0\\ 0 & 1 & 0 & 0 & -{W^{\prime }}_{1}\left(X_{gT}\right) & -{W^{\prime }}_{2}\left(X_{gT}\right) \end{array}\right]D=\left[\begin{array}{c} 0\\ 0 \end{array}\right]$$

The data selected in this paper are shown in Table 2.

## 3. Adaptive Augmentation Controller Design

The adaptive augmentation controller combines the adaptive controller with a classically designed linear control system using a multiplicative forward gain that enhances the system by adjusting the total loop gain in real time based on the error between the actual output and the output of the reference model. When the baseline controller performs well, the adaptive augmentation controller produces little enhancement. When the baseline controller is unable to effectively meet the performance requirements, the adaptive augmentation controller adjusts the total gain of the system by increasing/decreasing the adaptive gain to improve the performance of the control system; when the system is in a high degree of uncertainty or deviates from the nominal system, the adaptive controller can compensate the PD baseline controller to a greater extent to avoid system instability.

**Figure 1:**
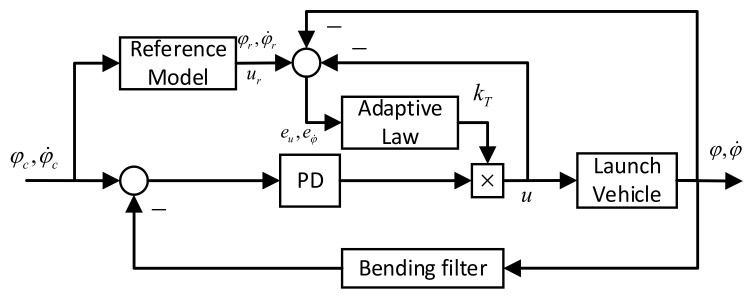
The adaptive augmentation control system.

Figure 1 shows the adaptive augmentation control block diagram, which mainly consists of two parts: the PD-based baseline controller and the adaptive controller composed of the reference model and the adaptive law.

### 3.1. Reference Model

The reference model was used to simulate the controlled motion of the rigid body of the launch vehicle in the nominal state, which produced the nominal response to the guidance instruction by adjusting the control parameters, and then the gap with the actual response of the launch vehicle was used as the input of the adaptive control law to adjust the gain of the PD controller. In adaptive gain control, a typical second-order system is used as the reference model [27], and the reference model used in this paper was obtained by neglecting the elastic vibrations in (5); the model’s state space is given in (8):

(8) $$\begin{array}{l} {\dot{x}}_{r}=A_{r}x_{r}+B_{r}u_{r}\\ y_{r}=C_{r}x_{r}+D_{r}u_{r} \end{array}$$

where $A_{r}=\left[\begin{array}{cc} 0 & 1\\ -b_{2} & -b_{1} \end{array}\right]$, $B_{r}={\left[\begin{array}{cc} 0 & -b_{3} \end{array}\right]}^{T}$, $C_{r}=\left[\begin{array}{cc} 1 & 0\\ 0 & 1 \end{array}\right]$, $D_{r}=\left[\begin{array}{c} 0\\ 0 \end{array}\right]$.

### 3.2. Baseline PD Controller

In the stability of elastic vibration, there is a difference between amplitude stability and phase stability. The so-called amplitude stability refers to the amplitude $Gain\left(\omega \right)<0$, when the phase-frequency curve crosses $\pm \left(2n+1\right)\pi$; the so-called phase stability refers to the amplitude $Gain\left(\omega \right)>0$, when the phase-frequency curve does not cross $\pm \left(2n+1\right)\pi$ The magnitude of stability of the essence of the engine oscillation control force generated by the excitation is less than the elastic vibration in the inherent damping under the role of attenuation, so the magnitude of stability depends on the inherent damping of elastic vibration and control system on the elastic vibration signal of the sufficient attenuation. The essence of phase stability is to take the elastic vibration signal as part of the control signal through the correction network to obtain the correct phase; for the elastic vibration to produce additional damping to achieve the purpose of stability, the phase stability does not depend on the inherent damping of elastic vibration, but the phase-frequency characteristics of the correction network put forward strict requirements.

**Figure 2:**
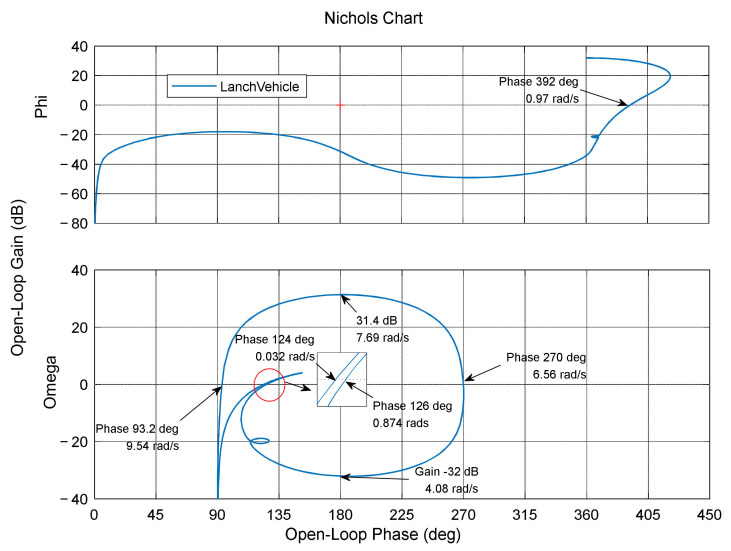
The open-loop frequency characteristics of the system.

Figure 2 shows an open-loop Nichols plot of the pitch channel, in which the open-loop frequency response of the angle (Phi) satisfies the amplitude-stability and phase-stability conditions, while the angle rate (Omega) does not satisfy the amplitude-stability condition (amplitude $Gain\left(\omega \right)>0$ when the phase frequency curve traverses the 180° curve) or the phase-stability condition (the phase frequency curve traverses the 180° line at frequency 7.694 rad/s, while amplitude $Gain\left(\omega \right)>0$). In adaptive augmentation control, the PD controller provides the basic control gain for the launch vehicle and is the basic controller in the AAC control framework. In this paper, we directly selected the PD controller parameters $K_{p}=-9.2$ and $K_{d}=-3.8$. For the rigid–flexible coupling model at the 30 s moment, the following notch filter was established as in (9):

(9) $$W\left(s\right)={\left(\frac{s^{2}+2\times 0.005\times 7.69s+7.69^{2}}{s^{2}+2\times 0.6\times 9s+9^{2}}\right)}^{2}{\left(\frac{s^{2}+2\times 0.005\times 7.69s+7.69^{2}}{s^{2}+2*0.6\times 6.8s+6.8^{2}}\right)}^{2}$$

**Figure 3:**
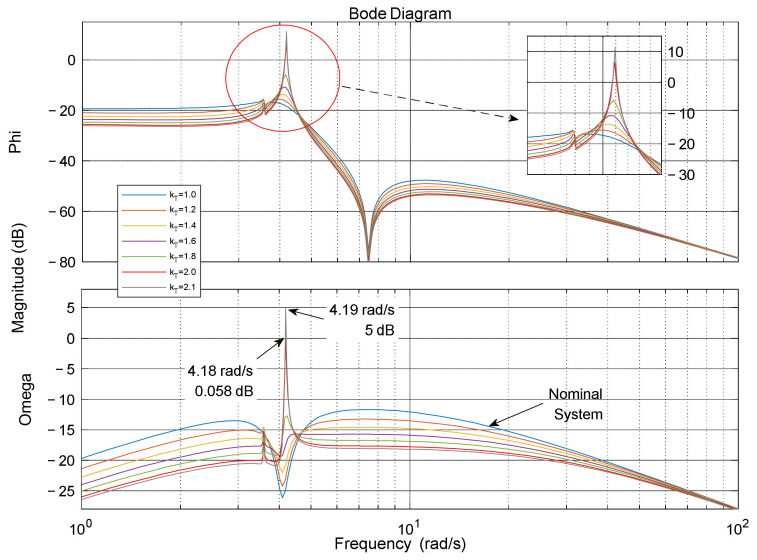
The adjustable range of forward gain.

In a classical PID feedback control system, a higher forward gain can improve the performance and robustness of the system with a fixed ratio of proportional and differential gain. However, due to the special performance requirements and stability requirements of large flexible launch vehicles (presence of high uncertainty, elastic vibration), forward gain must be limited to a small range to provide better performance and robustness for the baseline controller, and the design requirements usually limit the allowable forward gain to a range not less than 6 dB from the critical stability value to improve the system’s ability to cope with uncertainty. The closed-loop spectral characteristics of the system can be reflected to some extent by the open-loop margin of the system, and when the adaptive gain $k_{T}$ reaches a certain critical value, the closed-loop system exhibits resonance phenomena at certain frequencies (open-loop characteristics cross the jω axis in the complex plane), while for any $k_{T}+\varepsilon$, the closed-loop system exhibits dispersion phenomena. The spectral characteristics of the closed-loop system at different forward gain *K_T_* are shown in Figure 3. From this, the critical value of the octave forward gain in the adaptive augmentation control can be determined.

### 3.3. Multiplicative Adaptive Control Law Based on Spectral Damping

**Figure 4:**
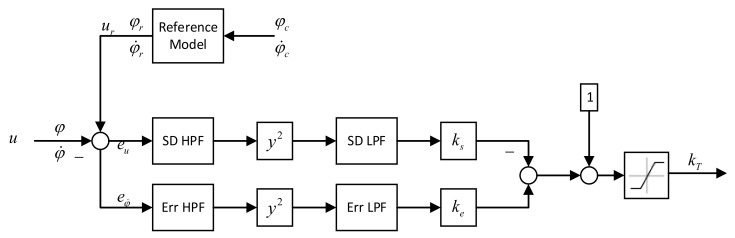
The adaptive control algorithm.

For large launch vehicle elastic vibration, the first-order elastic vibration mode has a low frequency and small phase deviation, and is usually stabilized by the phase-stabilization method, while the second-order and higher-frequency elastic vibration modes have large phase deviations, and are usually stabilized by the amplitude-stabilization method. In general, the first-order vibration mode can be phase-stabilized by selecting the mounting position of the rate gyro, the second-order vibration mode requires amplitude stabilization, and the higher-order vibration mode is amplitude-stabilized by high-frequency filtering of the correction network. Figure 4 shows a block diagram of the improved adaptive broadening control system designed in this paper, where the control command error signal $e_{u}$ and tracking error $e_{\varphi ,\dot{\varphi }}$ between the reference model and the actual model were used as inputs to the adaptive control method, and the adaptive gain $k_{T}$ was calculated through the two channels of oscillation suppression and error suppression, respectively, to adjust the baseline PD controller gain. The control command error, tracking error, and adjustment gain of adaptive augmentation control are shown below:

(10) $$e_{u}=u_{r}-u$$

(11) $$e_{\varphi ,\dot{\varphi }}=0.5e_{\varphi }+e_{\dot{\varphi }}=0.5(\varphi_{r}-\varphi )+({\dot{\varphi }}_{r}-\dot{\varphi })$$

(12) $$k_{T}=sat_{k_{0}}^{k_{\max}}\{k_{e}y_{e}-k_{s}y_{s}+1\}$$

where *k*_max_ is the upper bounds of the adjustment gain, *k*_0_ is the lower bounds of the adjustment gain, *k_e_* is the adjustment gain of tracking error term, *y_e_* is the output signal of the tracking error signal through the high- and low-pass filters, *k*_s_ is the adjustment gain of control command error term, and *y_s_* is the output signal of the control command error signal through the spectrum dampener.

### 3.4. Spectrum Dampers

The adjustment gains *k_e_* and *k_s_* of the two spectrum dampers adjust the spectrum output signals *y_e_* and *y_s_* of the two channels, which are formed by the tracking error signal and the controller command error signal, as follows:

(13) $$y_{e}=ErrL_{P}(s){\left(ErrH_{P}(s)e_{\varphi ,\dot{\varphi }}\right)}^{2}$$

(14) $$y_{s}=SDL_{P}(s){\left(SDH_{P}(s)e_{u}\right)}^{2}$$

In general, the DC gain of the designed high-pass filter should be as small as possible (the passband gain is usually set to 1), while the transition band should be as steep as possible (limited to 1.5 rad/s) The forms of the high- and low-pass filters are shown in (15) and (16):

(15) $$H_{P}(s)=\frac{s^{2}}{s^{2}+2\xi_{hp}\omega_{hp}s+\omega_{hp}{}^{2}}$$

(16) $$L_{P}(s)=\frac{\omega_{lp}{}^{2}}{s^{2}+2\xi_{lp}\omega_{lp}s+\omega_{lp}{}^{2}}$$

where $\omega_{hp}$, $\omega_{lp}$ are the cutoff frequencies of the high- and low-pass filters; and $\xi_{hp}$, $\xi_{lp}$ are the damping ratios, with values ranging from 0.5 to 0.8.

In the tracking error channel, the high- and low-pass filters successively process the error signal, and the AAC gain *k*_T_ is to be enhanced by increasing the error suppression gain *k*_e_, thus improving the overall forward gain of the system to reduce the system tracking error and improve system performance. In this channel, we set the control frequency near the shear frequency of the rigid-body system (0.87 rad/s), considering the need to compensate the control of the system tracking error and improve the overall gain of the system [5,28]. The shear frequency of the low-pass filter is near this frequency, and the shear frequency of the high-pass filter is one octave above this frequency, so the transfer function of the high and low-pass filters is given accordingly as follows:

(17) $$ErrH_{P}(s)=\frac{s^{2}}{s^{2}+2\times 0.8\times 8.7s+8.7^{2}}$$

(18) $$ErrL_{P}(s)=\frac{1.2^{2}}{s^{2}+2\times 0.6\times 1.2s+1.2^{2}}$$

In the control command error channel, the spectrum damper is mainly used to process the elastic vibration signal in the control command and reduce the AAC gain *k_T_* by setting a specific frequency to adjust the elastic rejection gain *k_s_*, thus reducing the overall gain (excessive gain) of the system to suppress the elastic vibration of the system and reduce its instability. The input of the high- and low-pass filters is the additional instruction error generated by the rigid-body controller instruction and the elastic vibration excitation, where the high-pass filter is used to obtain the elastic vibration signal from the control instruction. The analysis in the previous section showed that the system was prone to modal vibration at 7.69 rad/s, so we designed the control frequency at this frequency point. The cut-off frequency $\omega_{hp}$ of the high-pass filter should be taken slightly higher than this frequency. The low-pass filter is used to eliminate the high-frequency components of the signal, which is squared before entering the low-pass filter, so the value of the cut-off frequency $\omega_{lp}$ of the low-pass filter should be taken near this frequency. The corresponding parameters of the spectrum dampers in the elastic rejection channel are as follows:

(19) $$SDH_{P}(s)=\frac{s^{2}}{s^{2}+2\times 0.8\times 24.3s+24.3^{2}}$$

(20) $$SDL_{P}(s)=\frac{7.69^{2}}{s^{2}+2\times 0.6\times 7.69s+7.69^{2}}$$

## 4. Simulation Results and Analysis

**Figure 5:**
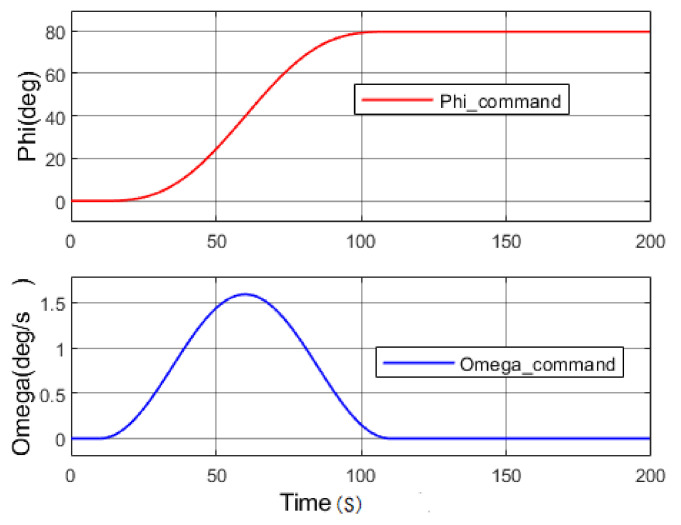
The pitch and angle rate commands.

In order to illustrate the role of the improved AAC control scheme in the launch vehicle system, the tracking curves and adaptive control gains of the nominal system and two different failure scenarios were presented and analysed in the simulation to verify the effectiveness of the designed algorithm. Assuming at 10 s after the launch vehicle takes off, the angle and rate commands of pitch were given as shown in Figure 5. The gain saturation function of the AAC controller in this example was taken as *K*_max_ = 2 and *K*_min_ = 0.5.

**Figure 6:**
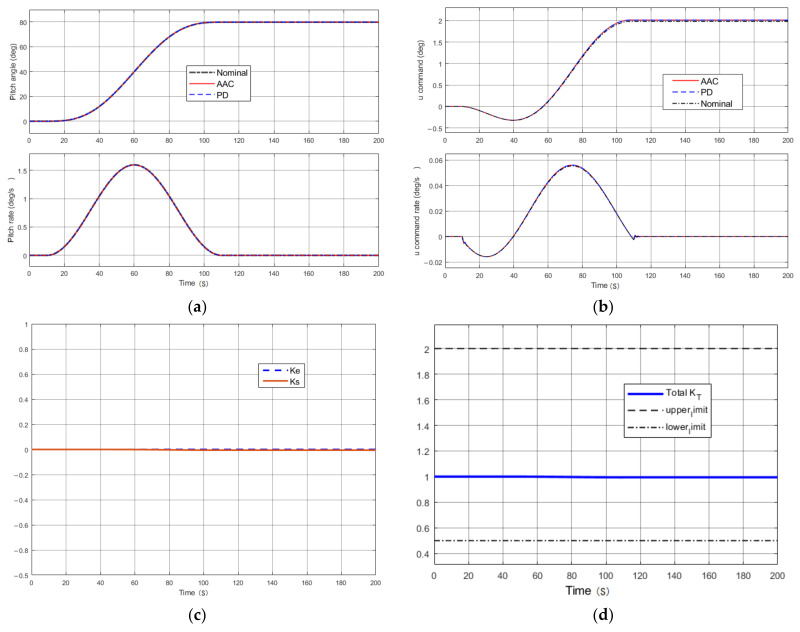
Performance in the nominal state: (**a**) pitching attitude; (**b**) control commands; (**c**) gain adjustment for the tracking error and control command error; (**d**) total gain of adaptive augmentation control.

If the system was in the normal state, the output of the system under PD control and AAC was consistent with the rigid-body nominal system, as shown in Figure 6a. The control commands and control rates of PD and AAC are shown in Figure 6b; there was almost no difference in the visible output curves. At the same time, the adjusted gain *k_e_* and *k*_S_ of two channels in the AAC were close to 0, as shown in Figure 6c, and the overall adaptive gain was always kept at a stable value *k_T_* = 1, which meant that the AAC did not produce any effect in the normal state. This was in line with our original design requirement that the AAC not be involved in control activities when the baseline PD controller was able to achieve a good performance output.

**Figure 7:**
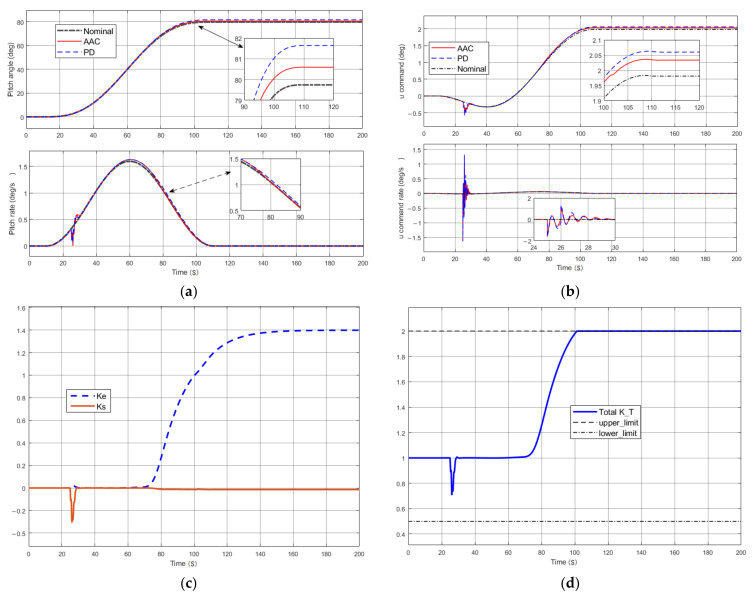
Performance in a state in which the baseline controller gain was misloaded and suffered external perturbations at *t* = 25 s: (**a**) pitching attitude; (**b**) control commands; (**c**) gain adjustment for the tracking error and control command error; (**d**) total gain of adaptive augmentation control.

We assumed that there was a baseline controller gain loading error during operation (the gain of the PD controller was not sufficient to meet the system requirements; in this case, $K_{p}=-1$ and $K_{d}=-0.8$); meanwhile, the control commands of the system were perturbed by a square wave with an amplitude of 0.5 and a duration of 5 s. As shown in Figure 7a, the system was able to track the input commands better, but then there was a certain steady-state error (about 2°) in the baseline PD control compared to the nominal system, and in contrast, the steady-state error was reduced to half (less than 1°) under the AAC control adjustment. In addition, in Figure 7b there is a corresponding reduction in the control command error, while the control command error rate only fluctuated significantly when it was just perturbed. Among the two channels, the adjusting gain *k*_s_ produced elastic suppression due to the disturbance in 25 s, and then the gain for suppressing elastic vibration fell back to 0; while *k*_e_ produced error suppression mainly after 60 s due to the tracking error. The overall gain *k*_T_ of the AAC control was less than 1 at 25 s in the elastic suppression channel *k*_s_, and then gradually increased (>1) due to the error suppression gain *k*_e_ generated, and the overall AAC gain was always maintained at a saturated value due to the long-term presence of steady-state errors.

**Figure 8:**
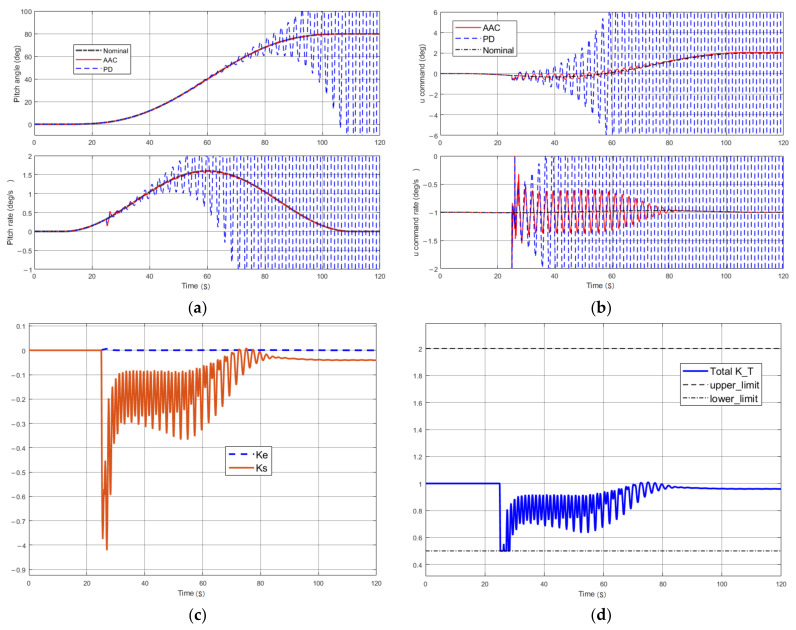
Performance at an elastic vibration frequency of 40% perturbation and external perturbation at *t* = 25 s: (**a**) pitching attitude; (**b**) control commands; (**c**) gain adjustment for the tracking error and control command error; (**d**) total gain of adaptive augmentation control.

Assuming that there was uncertainty in establishing the rigid spring coupling model, the elastic vibration frequency of the model was reduced by 40% while the same perturbation signals described above existed. The pitch angle and pitch angle rate signals at this time are shown Figure 8a. The system was able to follow the control commands to some extent in the early stages when the adaptive channel was closed (i.e., only the baseline PD controller was in action), but with the passage of time and accumulation, the system ended up in a divergent state. When the model parameters were changed substantially, the model was fundamentally changed, and the controlled object deviated from the nominal state. The original PD controller parameters were not suitable for this model, and the excessive forward gain aggravated the elastic vibration of the system. At the same time, the PD controller parameters were not reduced accordingly, which intensified the system oscillation and eventually could not be suppressed, leading to system dispersion. However, with the AAC controller, the system was able to suppress the system oscillation caused by the elastic modal perturbation and could track the reference input. At the same time, as shown in Figure 8b, for the control command and rate output, we can see that the control command of the baseline PD controller began to oscillate and could not be inhibited at the same time by the perturbation signal, while the AAC could well inhibit the oscillation of the control command (the control command error was less than 0.5, and the control command error rate was less than 1), which was beneficial for the actuator in the actual system, and had a good input signal.

The adjusted gain of the two channels *k*_e_, *k*_s_ and the overall gain *k*_T_ of the AAC control are shown in Figure 8c,d. We observed that in the case in which the AAC was involved in the control, the baseline PD controller was not able to achieve a good tracking effect due to the ingress of the elastic mode, then the AAC controller generated a corresponding gain value *k*_s_ (in this case mainly for the suppression of elastic vibration), and then set the AAC gain to less than 1 to reduce the overall gain of the system and meet the requirements. When the baseline controller can achieve the tracking effect better, then the value of AAC gain KT will fall back to 1. It is obvious from the above analysis that the adaptive control designed in this paper had a good robust stability to the ingress of the elastic mode, and under the adjustment of the AAC control, the launch vehicle could adjust the control gain online and in real time to set the engine swing angle of the servo to keep the rocket stable.

## 5. Conclusions

Adaptive augmentation control has important research significance and development potential for the control of large flexible launch vehicles, and can increase the robustness of the system, avoid oscillations and even destabilization problems caused by the estimation errors of the flexible mode, and improve the safety and reliability of rocket operation. The improved AAC control scheme designed in this paper had good performance, and the simulation showed that during the nominal state of the system, the AAC control did not affect the baseline PD controller. When the system was subjected to external disturbances or PD controller errors (the controller parameters were loaded at values less than the set value), the AAC control could generate a multiplicative gain greater than 1 to boost the system forward gain and reduce the steady-state error of the system to half of the PD control (<1°). With large regression of the flexible modal vibration frequency, the PD controller could cause the system to become unstable (uncontrollable). The AAC control could reduce the system forward gain by generating a multiplicative gain of less than 1 and limiting the control input signal to 0.5° to keep the system in a stable operation.

The simulation results of the above scenarios showed that the enhancement provided by the improved AAC control designed in this paper matched the expected goals and requirements, while the scheme had the same verifiability for the poorer control due to uncertainties caused by large-scale variations in thrust, mass, and atmospheric characteristics. The current simulation results showed suppression of up to 40% of the effect of flexible mode ingress, which greatly improved the robustness of the system. Future work on this program may consider sensor measurement noise and the nonlinear environment of closed-loop guidance [29], which would assist in fine-tuning and improving the extraction and calculation of error signals for use in conjunction with other load-shedding/fault-tolerant controls in the future development of large flexible launch vehicles.

## Figures and Tables

**Table 1:** Parameters and identification.

Notation	Identification
m	Mass of the launch vehicle
g	Gravitational acceleration
P	Total engine thrust
V	Arrow speed
$C_{y}^{\alpha }$	Lifting coefficient
q	Motive pressure
$S_{m}$	Characteristic area of arrows
θ	Trajectory inclination
α	Attack angle
σ	Heading (angle of course)
$J_{z}$	Moment of inertia
$\omega_{z}$	Pitch velocity
$m_{z}^{{\bar{\omega }}_{z}}$	Pitch damping torque factor
$\delta_{\varphi }$	Pitch channel motor pendulum
$x_{R}$	Distance from thrust point to arrow tip
$x_{z}$	Distance from mass centre to tip of arrow body
$x_{d}$	Distance from pneumatic core to arrow tip
l	Arrow length
$m_{R}$	Quality of each engine
$l_{R}$	Distance from engine pendulum to pivot
${\dot{W}}_{x}$	Radial apparent acceleration of rocket
$F_{y}$	Interference
$M_{z}$	Interference torque
$U_{iy}$	Micro-displacement of plane bending vibration
$R_{iy}$	Micro-element corner of distortion elastic vibration
$q_{iy}$	The *i*th-order oscillation pattern of the pitch channel
$\omega_{i}$	The *i*th-order oscillation angle frequency
$\xi_{i}$	The *i*th-order oscillation damping ratio

**Table 2:** Parameters and values.

Parameters	Values	Parameters	Values	Parameters	Values
$b_{1}$	0.6348	$D_{11}$	0.6407	$\omega_{1}$	3.5906
$b_{2}$	−0.0286	$D_{12}$	−27.2466	$\omega_{2}$	7.6941
$b_{3}$	1.1530	$D_{21}$	−1.6713	$\xi_{1}$	0.005
$b_{11}$	−3.2693 × 10^−5^	$D_{22}$	−3.6153	$\xi_{2}$	0.005
$b_{12}$	0.0015	$D_{31}$	−3.5752	$W_{1}{}^{\prime }\left(X_{T}\right)$	−0.015
$b_{21}$	7.2608 × 10^−4^	$D_{32}$	−142.71	$W_{2}{}^{\prime }\left(X_{T}\right)$	2 × 10^−4^
$b_{22}$	0.0029	$W_{1}{}^{\prime }\left(X_{gT1}\right)$	0.01	$W_{2}{}^{\prime }\left(X_{gT1}\right)$	0.004

## Data Availability

The data presented in this study are available on request from the corresponding author.
